# AGE-FRIENDLY PARTNER EVALUATION: REVIEWING FLORIDA ACTION PLANS

**DOI:** 10.1093/geroni/igad104.2359

**Published:** 2023-12-21

**Authors:** Helena Swanson

**Affiliations:** Central Connecticut State University, Bristol, Connecticut, United States

## Abstract

Understanding community collaboration is crucial to advance age-friendly community change (Greenfield, et al., 2022). This poster’s purpose is to provide evidence about age-friendly partner involvement to bring awareness to the extent that partnerships are collaborative and holistic at the age-friendly action plan stage. We conducted a qualitative, thematic analysis by reviewing Age-Friendly Florida Action Plans (N = 13) to code for partner involvement. The evaluation used the World Health Organization’s Age-Friendly Environments framework, which includes 4 cyclical steps: Engage and Understand, Plan, Act, and Measure. The evaluation questions were (1) How many partners are typically involved in action plans? and (2) Which types of partners are typically involved in action plans? The Florida Age-Friendly Communities evaluated have diverse characteristics in community type (e.g., rural, urban, and suburban), population size, and the percentage of the population that is 65+. After an iterative process was conducted between coders, a partner coding structure emerged. The partner coding structure included codes for government, businesses, non-profits, communities, and other partners. Partner themes for each sector (government, businesses, non-profit, community, and other partners) were identified and will be discussed. Overall, results concluded that there were an average of 16 partners involved in age-friendly action plans. On average, age-friendly partners were most involved in the Act step of the World Health Organization’s Age-Friendly Environments framework. Lastly, government sectors were the most frequent partner identified in age-friendly action plans. Considerations and recommendations for age-friendly partner involvement for creating collaborative and holistic age-friendly partners will be included.

